# Trace metal toxicity in some food items in three major markets in Ado-Odo/Ota LGA, Ogun State, Nigeria and associated health implications

**DOI:** 10.4314/ahs.v20i4.63

**Published:** 2020-12

**Authors:** Opeyemi Isaac Ayanda, Oluwakemi Adetutu Bello, Oluwatosin Ifeanyichukwu Nwabuisi

**Affiliations:** Department of Biological Sciences, Covenant University, Ota, Ogun State, Nigeria

**Keywords:** Cadmium, food items, lead, Manganese, nickel, zinc

## Abstract

**Background:**

Many of the markets in Nigeria are open, where foodstuffs are laid bare on flat trays and open baskets, directly exposing them to environmental contaminants. This study aimed at determining whether some food items on sale around an industrialized area of Ogun State are contaminated with trace metals.

**Methods:**

Seven different food items – *Clarias gariepinus* (roasted, fresh and smoked) *Bos taurus* (dried and fresh beef), zobo leaf (Hibiscus sabdariffa) and crayfish (*Astacus leptodactylus*) were sampled from three major markets, namely: Lusada, Atan and Ota in Ado-Odo/Ota LGA of Ogun State. They were analyzed for Lead, Cadmium, Nickel, Manganese and Zinc using Atomic Absorption Spectroscopy.

**Results:**

Cd was not detected in most of the food items across the markets. Pb, Ni and Mn were detected in very high concentrations above the maximum allowable limits by international regulatory agencies. Zn was the only metal that was generally below regulatory limits in food items across the three markets. The Estimated Daily Intake (EDI) and Target Hazard Quotient (THQ) values were generally higher than values acceptable in food.

**Conclusion:**

Some of the food items consumed in this area are not entirely safe from metal toxicity and this may have serious health consequences.

## Introduction

Trace metals are a metallic group associated with contamination and showing potential for ecotoxicity^1^. They do not degrade, they are indestructible and cannot be broken down into less harmful substances, making them accumulate easily in living tissues^2^. According to the World Health Organization^3^, this group of metals have been classified as risky when humans are exposed to them via food.

Fish, meat and fruit drinks are important for human diet because they provide the body with essential nutrient biomolecules such as vitamins, minerals, proteins. There is increasing concern about anthropogenic impacts on the ecosystem. The ability of metals to exert toxicity, bio accumulate and bio magnify in the food chain presents them as a serious threat to all forms of life^4^. Recently, efforts to determine the concentrations of metals in fish and other food items in order to determine their potential hazard to humans has been given much attention^5^.

The increase in the growth of industries worldwide, recently, have the capacity to affect weather, food and water quality^6^. In addition to the large quantities of waste released by food, chemical and textile industries, toxic substances are also discharged into the environment. Herbicides and chemical fertilizers appied on agricultural fields also contribute high amounts of pollutant-containing trace metals to atmospheric air pollution^7^.

Noxious metals bioaccumulate in organisms because they are hard to metabolize, they can bind to vital cellular components, such as structural proteins, enzymes, and nucleic acids, and interfere with their functions^8^. Symptoms and effects can vary according to the metal or metal compound, and the dose involved. Generally, long term exposure to toxic metals can have carcinogenic, circulatory, central and peripheral nervous system effects^9^. Some of these metals (Zn, Fe, Mn) are essential, enabling proper functioning of the human cells when present in certain range of concentrations within the cell and thus can impair important biological processes when deficient^10^. Furthermore, when present in the tissues at elevated concentrations, they can become toxic. On the other hand, other metals like Cd, Pb, Ni exert toxicity even at low concentrations and are termed non-essential. Cd is known to damage bones, kidneys and induce DNA damage^11^. It has also been identified as a potential human carcinogen, causing lung cancer. Pb exposures have developmental and neurobehavioral effects on fetuses, infants and children. It can also elevate blood pressure in adults^12^. Arsenic has also been reported to be implicated in both mutagenesis and carcinogenesis^13^.

A positive correlation exists between air pollution and food contamination^14^. Most of the food items (staple foods and snacks) on sale in Nigeria are poorly packaged and are displayed by the roadside^15^. These food items are seen as “ready-made” and are sold anywhere^16^. Wherever food is bought, it is important that its safety is assured. However, due to factors ranging from poor method of preparation and packaging, emissions from industries and automobile exhaust, improper disposal of wastes and pollution from other sources, these foods sold openly in the environment end up being contaminated^17^.

Assessing health risk to humans by estimating daily intake and hazard quotient is widely used by researchers to determine hazardous metals risk. It is an efficient way of assessing health risk levels posed by environmental pollutants^18^,^19^. Most metals enter the body through diet. Therefore, knowledge of metal concentrations in food products is vital in order to evaluate their hazard level in relation to the maximum residual limit for human consumption. Hence in this study, we compared the concentrations of selected trace metals in different food items sold in major markets in Ado-Odo/Ota, Ogun State, Nigeria, and further assessed the health risks associated with consuming these foods.

## Materials and methods

### Study Area and Location

Ado-Odo/Ota is one of twenty (20) local government areas in Ogun State, South-western, Nigeria. It is located on latitude 6°41′N to 6°68′N and longitude 3°41′E to 3°68′E. It is one of the local government areas in the Yewa zone of the state. Major towns in this area include Sango Ota, Iju-Ota, Owode, Igbesa, Atan, Agbara and Ado-odo. The population of the Local Government is about 14% of the entire state. Ado-Odo /Ota has a very rich concentration of industries, spread across the major towns in the local government. As much as there are industries, there are also major markets in these towns. Lusada (L), Ota (O) and Atan (A) were the three markets from which the sampling was done.

### Collection and treatment of Samples

A total of eighty-four samples of seven food items were collected within an eight week period, February - March 2018. The food items included *Clarias gariepinus* (roasted, fresh and smoked) *Bos taurus* (dried and fresh beef), zobo leaf (*Hibiscus sabdariffa*) and crayfish (*Astacus leptodactylus*). These food items were randomly purchased from different locations within the markets every two weeks (21 samples per fortnight), and this was done in all the three markets. All samples were brought in polythene bags to the laboratory for processing. In the laboratory, each food item was thoroughly washed with distilled water.

### Digestion and analysis of samples

Concentrated nitric acid and hydrochloric acid in the ratio 3:1 was added to 1g of each sample in a beaker. Digestion of the mixture was done at 100 °C until a clear solution was achieved. Solution was allowed some time to cool and then filtered to remove precipitates. Laboratory analysis of metals in the food items was conducted using the atomic absorption spectrometer (Perkin Elmer Atomic Absorption Spectrometer Pinnacle 900T, Perkin Elma, U.S.A). The metals analyzed include Pb, Mn, Zn, Ni and Cd. Blank was prepared for each sample on which adjustment was made by reference to blank. Accuracy of analytical procedure was ensured using certified reference material (DORM-3) after repeated sample analyses. As a form of precaution, all solutions were prepared using de-ionized water. All glass wares and plastic materials were soaked in 10% nitric acid and rinsed in de-ionized water before using. Results were converted to mg kg-1 dry weight of samples. Samples were spiked with different concentrations of the metals, containing known standards of all the metals tested for. Mixtures were digested and analyses was done in triplicates. Mean recovery percentages of between 85% and 95% were obtained for each of the metal, while limits of detection between 0.2 and 0.7 were detected for the metals. All reagents, HNO_3_ and HCl used were of analytical grade and 99% purity.

## Statistical analysis

### Metal concentrations

Mean values for the five measurements for each food item from each market were taken and subjected to analysis of variance (ANOVA) using GraphPad Prism software to determine whether or not differences were significant. Duncan multiple range test was used to compare means. Significant level was set at probability lower than 0.05 (p<0.05).

### Daily Intake Estimation

Estimation of Daily Intake (EDI) of metal consumption from these food items was calculated using the formula below

EDI = (MC x DIf/DIm/DIv) x BW-1 where MC is the concentration of metal detected from the sampled food item, DIf/DIm/DIv represents the average daily intake of fish, meat and vegetable in Nigeria, which were taken to be 0.10220, 0.1821 and 0.3522 kg/person/day respectively. Average body weight was taken as 60 kg. Average daily intake of vegetable was used for zobo drink because it is prepared from a leafy vegetable.

### Estimation of Target hazard quotient

Target Hazard Quotient (THQ) is the ratio between Estimated Daily Intake (EDI) and reference oral dose (RfD) normally used to estimate the non-carcinogenic risks associated with consuming these food items. The reference values for the metal were taken to be Zinc = 0.3, Manganese = 0.1423; Pb = 0.004, Ni = 0.02, Cd = 0.00124. The THQ value depending on whether it is greater or less than 1 can be used to infer likely adverse health effects. It is expressed below as THQ=EDI/RfD, where THQ is the hazard quotient and RfD is the reference dose (mg/kg/day).

## Results

The average values and the standard deviations of Pb, Mn, Zn, Ni and Cd concentrations in all the food items and in all the markets sampled are shown in tables 1 to 5. The concentration of lead in all the food items across the three markets varies (Table 1). Atan market had the highest concentrations of lead in dried meat, zobo leaf and roasted fish while Lusada market showed the highest lead concentrations in fresh fish, crayfish and smoked fish. Lead was not detected in the fresh meat sample from Ota and Atan markets. These differences were not all significant.

**Table 1:** Lead concentrations (mg/kg) in the food products from the three major markets

Food Products	Ota market	Atan Market	Lusada Market
Dried meat	61.66±1.86^a^	152.4±8.11^b^	103.01±7.31^ab^
Fresh meat	ND	ND	187.68±8.65
Zobo leaf	122.64±7.02^a^	215.96±11.25^b^	144.21±10.59^a^
Fresh fish	170.93±10.22^a^	92.02±8.04^b^	219.18±10.89^a^
Roasted fish	119.16±7.12^a^	151.73±9.36^a^	55.47±2.33^b^
Cray fish	61.92±2.05^a^	107.27±8.21^b^	165.2±9.48^c^
Smoked fish	125.72±7.55^a^	122.86±8.23^a^	137.33±10.56^a^

Mean±S.E, no significant difference in values with same superscript per row, where n = 4

**Table 2:** Nickel concentrations (mg/kg) in the food products from the three major markets

Food Products	Ota Market	Atan Market	Lusada Market
Dried meat	25.67±1.32^a^	57.23±1.86^a^	63.48±1.75^a^
Fresh meat	ND	75.12±2.05^a^	28.07±1.54^b^
Zobo leaf	52.16±1.71^a^	56.86±1.60^a^	73.37±1.85^a^
Fresh fish	57.72±1.62^a^	37.17±1.23^a^	38.76±1.46^a^
Roasted fish	65.96±2.04^a^	58.89±1.79^a^	45.63±1.52^a^
Cray fish	67.35±1.31^a^	46.64±1.06^a^	48.53±1.95^a^
Smoked fish	62.92±1.44^a^	47.88±1.26^a^	50.71±1.13^a^

Mean±S.E, no significant difference in values with same supercript per row, where n = 4

**Table 3:** Cadmium (Cd) concentrations (mg/kg) in the food products from the three markets

Food Products	Ota Market	Atan Market	Luasada Market
Dried meat	19.65±1.55	ND	ND
Fresh meat	13.31±1.24^a^	ND	3.75±0.21^b^
Zobo leaf	ND	ND	ND
Fresh fish	ND	ND	ND
Roasted fish	ND	ND	ND
Cray fish	ND	ND	ND
Smoked fish	ND	31.76±1.89	ND

Mean±S.E, no significant difference in values with same superscript per row, where n = 4

**Table 4:** Manganese (Mn) concentrations (mg/kg) in the food products from the three markets

Food Products	Ota Market	Atan Market	Lusada Market
Dried meat	17.13±0.65^a^	ND	47.37±2.44^a^
Fresh meat	81.1±2.87^a^	ND	14.42±1.06^b^
Zobo leaf	2.89±0.12^a^	4.18±1.66^a^	8.78±2.76^a^
Fresh fish	ND	84.80±2.59	ND
Roasted fish	76.04±2.68^a^	80.45±2.75^a^	73.45±2.04^a^
Cray fish	10.49±1.23^a^	62.58±2.74^b^	ND
Smoked fish	ND	ND	ND

Mean±S.E, no significant difference in values with same superscript per row, where n = 4

**Table 5:** Zinc (Zn) concentrations (mg/kg) in the food products from the three markets

Food Products	Ota Market	Atan Market	Lusada Market
Dried meat	111.84±5.76^a^	60.54±2.12^b^	45.27±2.01^b^
Fresh meat	70.54±3.10^a^	22.56±1.46^b^	33.37±1.12^b^
Zobo leaf	89.52±2.55^a^	13.33±1.10^b^	16.34±1.06^b^
Fresh fish	11.34±1.21^a^	3.13±0.13^a^	21.49±1.48^a^
Roasted fish	17.58±1.42^a^	22.85±1.33^a^	9.96±1.03^a^
Cray fish	63.05±1.58^a^	67.15±1.98^a^	23.64±1.31^a^
Smoked fish	55.36±2.45^a^	17.47±1.52^ab^	4.32±0.25^b^

Mean±S.E, no significant difference in values with same supercript per row, where n = 4

Nickel concentrations in all the food items across the three markets were not significant except in Lusada market (table 2). As presented in table 1, nickel was also not detected in fresh meat in Ota market. In Atan market, nickel was highest only in fresh meat. The highest concentrations of nickel in fresh fish, roasted fish, crayfish and smoked fish were all from Ota market, while nickel concentrations in zobo leaf and dried meat were observed in Lusada market. Only fresh meat showed any significant difference in nickel concentrations among the three markets. The least concentration of nickel (25.67±1.32) in all food items from all the sampling areas was observed in Ota market.

Table 3 shows the concentrations of cadmium in the food samples from the markets. Cadmium was not detected in majority of the food items from all the three markets. It was detected only in fresh and dried meat in Ota market, only in fresh meat from Lusada market and only in smoked fish from Atan market. There was a significant difference between the values observed in fresh meat from Ota and Lusada markets.

The average concentrations of manganese in all the food items across the three sampling areas is as presented in table 4. Not all the food items were positive for manganese. In all the markets, manganese was not detected in smoked fish. In Ota market, it was not detected in fresh fish and it was not detected in dried and fresh meat from Atan market. It was not detected in fresh fish and crayfish from Lusada market. Only in zobo leaf and roasted fish was it detected in all three markets. The highest concentration of manganese was observed in fresh fish from Atan market while the lowest was in zobo leaf from Ota market. Differences in the average concentrations of manganese were not all significant across the three locations.

As against what was observed for manganese, where it was not detected in all the food items, the other essential metal, zinc was detected in all the food items across the three sampling areas (table 5). The varying average concentrations of zinc are as presented in table 5. Dried meat has the highest concentration of zinc of all the food items, and this was observed in Ota market. The highest concentrations of zinc in fresh meat and zobo leaf was also from Ota market, roasted fish and crayfish from Atan market, and fresh fish from Lusada market.

Table 6 shows the reference values by different International agencies against which concentrations of the metals tested can be compared. On the basis of this comparison, the concentration of metals as reported in this study are high.

**Table 6:** Permissible limits of heavy metals in some foods

	Zn	Cd	Pb	Ni	Mn	Ref
Fish/Meat	-	0.05	0.1–0.3	-	-	FSAI^25^
	5	0.05	0.2	-	5.5	FAO^26^
Fruits/Drinks	0.3	0.003	0.01	0.02	0.2	SON^27^
	-	0.003	0.01	0.07	-	WHO^28^

The estimated daily intake for an adult of 60kg exposed to these metals is presented in table 7. Zn varied between 0.005 mg/kg b.w. per day in fish at Atan to 0.519 mg/kg b.w. per day in vegetables at Ota. As reported above, Cd was detected only in beef in Ota and Lusada markets. The EDI value was observed to be 0.011 mg/kg b.w. per day in the latter and 0.039 mg/kg b.w. per day in the former. The lowest EDI value for Pb was 0.156 mg/kg b.w. per day in Atan market and the highest value of 0.840 mg/kg b.w. per day was observed Lusada. The EDI for Ni ranged from between 0.304 mg/kg b.w. per day in Ota to 0.426 mg/kg b.w. per day in Lusada. Mn, the only other essential metal had values ranging from 0.009 mg/kg b.w. per day in Ota to 0.143 mg/kg b.w. per day in Atan market.

**Table 7:** Estimated Daily Intake of Metals (mg/kg b.w. per day)

Location	Food Items	Zn	Cd	Pb	Ni	Mn
Ota	Fish	0.019	-	0.289	0.097	-
	Beef	0.21	0.039	-	-	0.138
	Vegetable	0.519	-	0.712	0.304	0.009
Atan	Fish	0.005	-	0.156	0.063	0.143
	Beef	0.066	-	-	0.225	-
	Vegetable	0.076	-	0.366	0.327	0.024
Lusada	Fish	0.036	-	0.372	0.065	-
	Beef	0.099	0.011	0.561	0.084	0.042
	Vegetable	0.093	-	0.840	0.426	0.047

Table 8 shows the THQ for adults exposed to food contaminated with metals. Only Zn in vegetable from Ota market and Mn in fish from Atan market were above 1. The THQ value of these non-essential metals were below 1 in all the other food items. All the metals had THQ values that are very much higher than 1. Their values ranged from 11 -39 (Cd); 39 – 210 (Pb) and 3.15 – 21.3 for Ni.

**Table 8:** Target hazard quotient (THQ) for adults exposed to food items contaminated with trace metals in the study area

Location	Food Items	Zn	Cd	Pb	Ni	Mn
Ota	Fish	0.06	-	72.25	4.85	-
	Beef	0.7	39	-	-	0.99
	Vegetable	1.73	-	178	15.2	0.06
Atan	Fish	0.017	-	39	3.15	1.02
	Beef	0.22	-	-	11.25	-
	Vegetable	0.25	-	91.5	16.35	0.17
Lusada	Fish	0.12	-	93	3.25	-
	Beef	0.33	11	140	4.2	0.30
	Vegetable	0.31	-	210	21.3	0.34

## Discussion

This study was designed to analyze the concentrations of trace metal in various food items, mostly fish and meat in different forms from major markets in the Ado-Odo/Ota area of Ogun State. While there may have been reports of the accumulation of metals in fish, these reports are mainly on trace metals in fish from a particular water body, different fish species or aquatic products from the same body of water, the same fish species from different water bodies or different fish species from different water bodies. Humans have different preferences for the form in which they consume fish and meat. While some like both fresh, others prefer it smoke-dried, sun-dried, and some want it roasted. Since the processing of these fish and meat products varies, their ability to accumulate trace metals may not be the same. To the best of our knowledge, there is no individual reported study that compares the concentration of trace metals in the food items we sampled in this study.

The four highest lead concentrations are 219.18±10.89 (fresh fish, L), 215.96±11.25, (zobo leaf, A), 187.68±8.65 (fresh meat, L) and 170.93±10.22 (fresh fish, O). This means that three of these high values are from fresh fish and meat alone. This may probably mean that the fresh products have capacity to accumulate the metal as compared with drier fish and meat products irrespective of location. Only in roasted fish (55.47±2.33) and dried meat did the lead concentrations recorded in this study not have the highest values in Lusada market, even though the difference between the values for dried meat in Atan and Lusada are not significantly different. Among the three markets, Lusada market is the closest to the road side. Furthermore, apart from industries and institutions of learning being concentrated there, a lot of construction works are ongoing. According to Tiwari et al.^29^, most of lead concentrations found in the environment are as a result of anthropogenic activities. The pressure of vehicular movements and the use of construction equipment may have constantly exposed all these food items to lead, since they are openly displayed. Farouk et al.^30^ also reported high concentrations of lead in meat sold in markets.

The average concentration of lead in zobo drink is also very high, being significantly higher in Atan market compared to Ota and Lusada. Zobo is a local drink consumed in Nigeria. It is made by filtering the extract obtained from boiling the dried calyx of Hibiscus sabdariffa in water. The calyx of the *H. sabdariffa* contains numerous phytochemicals^31^. Besides enjoying a wide popularity in Nigeria, it is also used as a cold drink in other parts of the world like Egypt, America, Mexico, West Indies and Jamaica^32^. The dried calyx is usually sold in open markets. Bakare-Odunola and Mustapha^33^ and Maigari et al.^34^ both reported the presence of lead and other metals in zobo drink from different parts of Nigeria. However, the concentrations reported in this study is much higher than those reported by these authors. This is probably because lead was investigated directly in the already made zobo drink while it was the calyx used for analysis in this study.

Although nickel concentrations in all three markets and in all the food items are high, the differences are not significant (table 2). Nickel is a naturally occurring element, existing in different forms^35^. It is one of the various trace metals that are widely distributed in the environment, from both natural sources and anthropogenic activities, in addition to input from mobile and stationary sources. It is present in all environmental media - air, soil, water and soil^36^,^37^. Any of these sources may sufficiently explain the high nickel concentrations detected in these food items (Table 2). In addition, nickel and its compounds have been reported to have many industrial and commercial uses, with the growth of industries contributing significantly to its release into the atmosphere^35^. As earlier stated, the sampling area is a highly industrialized area, so this may also have contributed to the levels of nickel reported in this study. As seen in table 3, cadmium was not detected in most food items across all the sampling areas. Cadmium is a toxic metal that is present in tobacco smoke, air, water and other media. It is possible that the non-detection of cadmium in most of the food items is due to the generally low level of cadmium in the air and probably not too many tobacco smokers around the market area^38^.

Manganese and Zinc are both essential metals. Manganese usually occurs in the solid form in all environmental media. However, some of them can easily dissolve in water or be suspended in the air as small particles. Within a few days, small dust particles in the air will usually settle depending on weather conditions, their size, density and weight^39^. The varying concentrations of manganese in table 4 shows that zobo drink may be a particularly good food item as manganese supplement considering its concentration across the three markets. The non-detection of manganese in smoked fish might be attributed to the processing of the fish to dryness^40^. Differences in concentration across the three markets may be due to differences in the activities that releases manganese into the atmosphere.

Zinc was found to be present in all the food items across all the markets, though significant differences was not observed in all cases among the food items in the three markets. Human activities that release Pb into the environment has also been implicated in zinc environmental pollution. Traffic pollutants include metals as zinc and lead that are potentially toxic to the health of people^41^,^42^. High concentrations of zinc in food samples sold in different markets in Abeokuta, Nigeria was reported by Lanre-Iyanda and Adekunle^15^.

All the metals tested for in this study were above maximum allowable limits in fish, meat or drinks (Table 6). The EDI values for these metals exceeds limits set by USEPA^43^. Furthermore, the THQ of greater than 1 for all the metals is an indication of the likelihood for adverse effects from consuming these food items. This perhaps is a very serious cause for concern, considering the health implications. Lead has the capacity to cause damage to most of the human body systems - endocrine, skeletal, nervous, immune systems, circulatory and metabolic^44^. Furthermore, pregnant women, children, and the aged are more sensitive to lead exposure, while lead also significantly affects intelligence quotients and hampers children's physical development^44^.

Cadmium is one of the most toxic elements in the environment to which people can be exposed. It is not effectively excreted from the human body; rather, it accumulates. It also causes demineralization of the bones, whether by directly damaging the bone or indirectly through kidney dysfunction. Impairment of lung function and vulnerability to lung cancer are other effects that have been reported^45^.

Nickel exposure also has its side effects, even though it has been reported to have functional biological roles in organisms^46^. Lung fibrosis, skin allergies, iatrogenic nickel poisoning and cancer of the respiratory tract are some of the varying pathological conditions that may arise from human exposure to nickel^47^,^48^. Studies have also shown that nickel exerts hepatotoxic, nephrotoxic, haematologic and fetotoxic effects in addition to causing proteinuria and aminoaciduria^49^,^50^.

Mn is important to man's health, acting as a co-factor of various enzymes, and is required for normal development, maintenance of nervous and immunological functions, and regulation of blood sugar and vitamins^51^,^52^. However, over-exposure to manganese can result in permanent neurological damage^53^. There is evidence supporting the view that Mn exposure alters cardiovascular function significantly^54^. Mn exposure impairs myocardial contraction, dilates blood vessels, and can lead to hypotension.

Zinc like manganese is needed by the human body. It is a vital component of different enzymatic systems and zymoexcitators. It is needed for growth, helps to regenerate tissues and boosts the immune system. However, excessive zinc intake may lead to stomach cramps, nausea, and vomiting; exposure to high dosage of Zn in the long term can cause an imbalance in cholesterol, reduce the function of the immune system and can even decrease fertility^44^.

## Conclusion

Significant data on the concentration of trace metals in food items regularly consumed by man in the study area is reported in the present study. The high values reported for all the metals, especially the toxic metals, is a reflection of the level of contamination in the study area. Of further concern, is that the THQ of these toxic metals is greater than 1. The associated risk involved in consuming these food items should not be discountenanced. Consequently, this report creates an awareness on the need for persons to be cautious when buying fish and meat products from these markets, as they may not be safe for consumption.

## Figures and Tables

**Fig 1:**
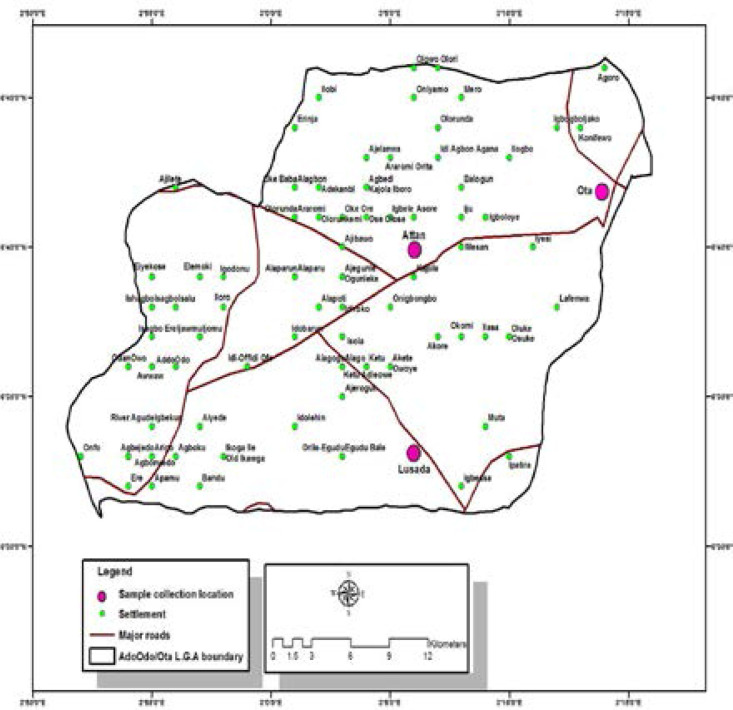
Map of Ado-Odo/Ota Local Government Area showing the Sampling Locations
